# Clinical Trials to Gain FDA Approval for Computerized Cognitive Training: What Is the Ideal Control Condition?

**DOI:** 10.3389/fnagi.2016.00249

**Published:** 2016-11-02

**Authors:** Jeffrey N. Motter, Davangere P. Devanand, P. Murali Doraiswamy, Joel R. Sneed

**Affiliations:** ^1^The Graduate Center, City University of New YorkNew York, NY, USA; ^2^Queens College, City University of New YorkNew York, NY, USA; ^3^Division of Geriatric Psychiatry, Columbia University and the New York State Psychiatric InstituteNew York, NY, USA; ^4^Duke Medicine and Duke Institute for Brain Sciences, Duke UniversityDurham, NC, USA

**Keywords:** cognitive training, active control, placebo, mild cognitive impairment, engagement

The explosive growth of mobile technologies combined with the rapid rise of aging populations fearful of their risk for Alzheimer's disease has led to a number of marketed products aimed at enhancing cognitive health. However, an increasing number of product claims that are not substantiated has led regulatory agencies, such as the Federal Trade Commission (FTC), to issue warnings or penalties against some companies. Therefore, it is likely that a number of computerized cognitive training (CCT) companies will conduct clinical trials to prove their efficacy to gain Food and Drug Administration (FDA) clearance as a medical software/device. This raises a number of issues such as optimal trial design for establishing efficacy. The type of control condition is unique issue for CCT, given the variety of non-specific known to produce beneficial effects on cognition that are difficult to isolate from the content of the program. These include participant expectancy, engagement, motivation, novelty, and therapist interaction. We herein discuss the types of non-specific factors, desirable qualities of an active control condition, and the nuances that exist between previously used control conditions within the context of CCT for mild cognitive impairment.

## Expectancy

One nonspecific factor is expectancy, which refers to the participant's anticipation of positive or negative treatment effects. Expectancies come in two major forms: outcome expectancy, which is the belief that the treatment itself will result in a particular outcome, and response expectancy, which is the participant's subjective response to the treatment. Expectancies of both kinds impact outcomes across modalities, including psychotherapy (Goossens et al., 2005; Smeets et al., 2008; Weinberger, 2014), pharmacological interventions (Rutherford et al., 2010), and neurosurgery (Freeman et al., 1999). Long known to produce clinically significant outcomes, the belief that one will symptomatically improve is met with high response rates. For example, a meta-analysis of randomized placebo-controlled trials of antidepressants found the placebo effect to be responsible for the majority of observed change, in depression symptoms, with a response rate of 50% for those receiving medications compared to 30% for groups assigned to the placebo condition (Walsh et al., 2002).

Further evidence for the contribution of expectancy to treatment effectiveness can be found in the different response rates across different trial designs. Greater response rates have been observed in comparator trials between two drugs than in placebo-control trials in late-life depression (Sneed et al., 2008). This discrepancy may be a consequence of participants in comparator trials knowing they will receive treatment, while participants in placebo-controlled trials are hoping they are assigned to the treatment arm (and hence have lower expectations). This finding has been extended in middle-aged and adolescent depression (Rutherford et al., 2011) as well as in schizophrenia (Rutherford et al., 2014). Although these groups are diverse in terms of presenting ailment, consistent among them is that degree of expected improvement coincides with magnitude of actual improvement.

Rutherford et al. (2010) elaborated on a model of expectancy effects driving the differences in response rates between types of trials in which the orbitofrontal cortex (OFC), rostral anterior cingulate cortex (rACC), and nucleus accumbens (NAC) generate and maintain expectancies (Rutherford et al., 2010). The OFC and rACC are active during anticipation of pain and unpleasant experiences (Petrovic et al., 2005). Placebos lower activation of these regions which coincides with reduced severity of experienced pain (Wager et al., 2004). This analgesic effect may be partially explained by heightened opioid activity in the OFC and rACC that follows placebo administration and correlates with reported pain alleviation (Wager et al., 2007). Activation of the NAC, on the other hand, occurs in anticipation of reward. Greater activation of the NAC occurs with expectancy of analgesia and is correlated with placebo-induced pain reduction (Scott et al., 2007). Taken together, heightened expectancy of relief attenuates appraisal of negative experiences to a clinically significant degree.

In addition to altering perception of negative symptoms, raising expectancy increases participant's belief of self-efficacy in their functioning, making them more confident in their own capability to perform tasks. Judgments of high self-efficacy are associated with greater exerted effort in the face of challenges, as well as lengthened persistence (Bandura, 1982). A systematic review of placebo responses found self-efficacy and locus of control to be significant predictors of symptom improvement (Horing et al., 2014). The impact of response expectancy on cognition is particularly important in trials of CCT, given the use of performance-based outcome measures. In trials of CCT, participants are told that improvement in their cognitive functions is possible as a benefit from their involvement in the study. This creates an expectation of better performance following training, which is known to create positive cognitive outcomes. Indeed, participants who enrolled in a trial of CCT via a flyer suggesting CCT will improve working memory and fluid intelligence had greater post-training performance than participants who enrolled via a non-suggestive flyer, despite participating in the same program (Foroughi et al., 2016). Similarly, in a trial of healthy adults randomly assigned to a placebo pill or no-pill condition, those who took the placebo pill described as a “cognitive enhancer” had greater performance on tests of attention and delayed recall, though no effect was found in five secondary outcome measures (Oken et al., 2008). Given the potential effect of expectancy on cognitive performance, and the wide use of neuropsychological tests as primary outcomes in CCT trials, treatment, and comparison conditions must be balanced in terms of anticipated improvement.

## Engagement

How well a CCT program captivates and sustains attention will affect the capability of subjects to participate and focus during training. How engaging a task is depends on numerous factors, including usability, focused attention, positive affect, esthetics, endurability, richness, and control (Wiebe et al., 2014). These characteristics can be independent of the main program content. For example, a 2-back task necessitates working memory ability by nature of the design alone. By adding auditory feedback, sound effects, colorful animations, score tracking, and countdown timers, the task becomes more attractive to the participant. Such design elements create a nonspecific cognitive load, are not unique to CCT, yet make the CCT condition more interesting than the comparison group. When comparing an engaging CCT task to either waitlist or uninteresting control activities, it becomes impossible to isolate the central treatment effect.

## Motivation

Whereas engagement may be understood as emotional or attentional investment during a task, motivation is a global personal orientation to the activity (Wiebe et al., 2014). A participant may believe that cognitive improvement is contingent upon active involvement in their training. Numerous cognitive training platforms track then share performance data with participants. Following conclusion of a module, participants are shown their score, typically alongside a report of previous attempts. Congratulatory messages for surpassing earlier records are common. This positive feedback and potential for goal-setting behavior creates an environment where the participant is motivated to perform well.

Participant's beliefs about the malleability of their intelligence will affect their performance as well. In a study evaluating performance on tests of general knowledge, participants who held the believe that intelligence is malleable corrected more errors in their responses during retesting than participants who believed that intelligence is fixed (Mangels et al., 2006). When given negative feedback following errors, participants who believed in fixed intelligence demonstrated reduced memory-encoding activity in the temporal lobe. This suggests that one's attitude toward learning will affect the amount of effort placed in training tasks. This is particularly important for older adults with MCI, whose motivation toward the task may be increased by the prospect of preventing further cognitive decline.

## Novelty

The novelty effect is the tendency for improved performance due to interest in a fresh experience, rather than the content. A review of educational research found that when not controlled, novelty effects create an increase in test scores. On average, performance increases due to novelty by 50% of a standard deviation for the first 4 weeks, and by 20% of a standard deviation after 8 weeks (Clark and Sugrue, 1991). Novel experiences may also simply be remembered better than familiar ones. On tasks of explicit recognition, subjects show higher accuracy in identifying novel words from a studied list than familiar words viewed multiple times (Tulving and Kroll, 1995; Habib et al., 2003). Given that CCT consists of tasks not encountered in everyday life, and prior familiarity with training protocols is grounds for excluding participants, there is ample opportunity for sheer novelty of the task to constitute a considerable portion of any measured effects.

## Active controls

To address these issues, studies have employed active controls, where the comparison condition completes an activity designed to account for non-specific factors. This type of control condition aims to determine whether the benefits of such mental exercise are unique to CCT or can be obtained by any stimulating activity. Commonly used active controls include crossword puzzles, word searches, newspapers, and questionnaires. Active controls alleviate ethical issues of placebo-controlled treatments, as both the treatment and comparison condition may expect improvement. Although active controls are methodologically superior to waitlist conditions, differences in effect sizes between active control conditions and passive control conditions are not consistently observed (Karbach and Verhaeghen, 2014; Au et al., 2015), and the nature of the active control task may not account for all non-specific effects. Given the diversity of CCT paradigms and quantity of non-specific factors, researchers have argued that there is no universally applicable active control condition for all design cases (Boot et al., 2013). Instead, the control condition must be reasonably related to the tasks of the CCT condition such that participants expect improvement in the same cognitive domain regardless of their group assignment.

Table 1 contains a list of studies of CCT in patients with mild cognitive impairment (MCI). Most studies use waitlist control conditions (Rozzini et al., 2007; Finn and McDonald, 2011) or control conditions that do not account for engagement and motivation in the task (Galante et al., 2007; Talassi et al., 2007; Gagnon and Belleville, 2012; Herrera et al., 2012; Carretti et al., 2013; Gaitan et al., 2013). In such designs, the treatment condition is at an unfair advantage because patients assigned to CCT have a greater chance of improvement simply because the tasks are engaging and motivating whereas those assigned to waitlist control lose interest and motivation. Even when active control conditions have been used in previous studies of CCT in MCI, they are not consistently computerized (Talassi et al., 2007; Herrera et al., 2012; Carretti et al., 2013; Gaitan et al., 2013) and consist of CCT tasks of invariable complexity (Gagnon and Belleville, 2012; Gaitan et al., 2013). Comparing CCT that scales in difficulty with participant performance to CCT that remains at a fixed difficulty does not determine whether effects are specific to CCT or can be obtained with any engaging computerized game. Further, trials of scaling difficulty fail to take into account the novelty of experience they introduce, beyond just harder problems.

**Table 1:** **Studies of CCT for MCI**.

**Study name**	**Treatment condition**	**Control condition**	**Control Weaknesses**
Barnes et al., 2009	Processing speed and auditory processing CCT (*n* = 22)	Audio books, reading online newspapers, visuospatial computer games (*n* = 25)	Heterogeneous control tasks, not matched for engagement
Carretti et al., 2013	Working memory CCT (*n* = 10)	Memory discussion and questionnaires (*n* = 10)	Control group not computerized, unequal socialization opportunities
Finn and McDonald, 2011	Attention, processing speed, visual memory, and cognitive control CCT(*n* = 12)	Waitlist (*n* = 13)	Non-active control condition
Gaitan et al., 2013	CCT + pen and paper cognitive training (*n* = 37)	Pen and paper cognitive training (*n* = 23)	Control group not computerized, not matched for engagement
Galante et al., 2007	Attention, memory, language, abstract reasoning, and visuo-spatial CCT (*n* = 7)	Life history and current event discussion (*n* = 4)	Heterogeneous control tasks, not matched for engagement, not computerized, small sample
Gagnon and Belleville, 2012	Variable priority divided attention tasks (*n* = 13)	Fixed priority variable attention tasks (*n* = 13)	Does not account for engagement, novelty
Gooding et al., 2016	Milieu-based attention, memory, and executive function CCT (*n* = 23) Attention, memory, and executive function CCT (*n* = 31)	BrainAge, Sudoku, crosswords (*n* = 20)	Heterogeneous control activities, different CCT vs. control interface
Herrera et al., 2012	Short-term memory CCT (*n* = 11)	Fact searching, crossing out letters, sentence construction (*n* = 11)	Heterogeneous control tasks, not entirely computerized, not matched for engagement
Rosen et al., 2011	Processing speed and auditory processing CCT (*n* = 6)	Audio books, reading online newspapers, visuospatial computer games (*n* = 6)	Small sample size, heterogeneous control tasks, not matched for engagement
Rozzini et al., 2007	Attention, memory, language, abstract reasoning, and visuo-spatial CCT + cholinesterase inhibitors (*n* = 15)	Cholinesterase inhibitors only (*n* = 22) And Waitlist (*n* = 22)	Non-active control condition
Talassi et al., 2007	CCT, occupational therapy, and behavioral training (*n* = 54)	Physical rehabilitation, occupational therapy, and behavioral training (*n* = 13)	Heterogeneous control tasks, not matched for engagement, not computerized

The CCT conditions themselves vary considerably, both by targeted cognitive domains and whether they are administered alone (Galante et al., 2007; Barnes et al., 2009; Finn and McDonald, 2011; Rosen et al., 2011; Gagnon and Belleville, 2012; Herrera et al., 2012; Carretti et al., 2013; Gooding et al., 2016), or as part of a wider intervention (Rozzini et al., 2007; Talassi et al., 2007; Gaitan et al., 2013). If CCT is administered alongside additional therapies, the magnitude of participant's expectancy, engagement, and motivation may be greater than if they were treated with CCT alone. Such nuances in design will impact the magnitude of the observed differences between the training and control groups, and the measured effect sizes across trials. One needs to consider the nonspecific factors present in both the CCT and control conditions in order to accurately interpret the results.

## Model control conditions

An adequate comparison condition must be matched for engagement, motivation, training time, computer interface, and novelty of stimuli. Preferably, the control group should account for nonspecific factors without introducing neurocognitive demands. If it does create specific effects, their impact on cognitive functioning should be known before comparing it to a CCT group. By using a CCT group, an active control group, and a passive control group, the relative contributions of each nonspecific factor can be estimated (Greenwood and Parasuraman, 2016). There is no “one size fits all” control condition. Instead, the selection of a control condition must be made in view of the content of the CCT platform and the specific research question.

An example of a well-balanced active control condition is a CCT program targeting domains of cognition that are different from those being targeted in the CCT program of the intervention condition. Should one group find greater improvement in cognitive domains of interest that transfer to untrained domains and quality of life, this can be taken as evidence that the content of CCT matters more than the non-specific factors. Participants in the active control group must expect the same types of cognitive benefits as those in the training group, principally by the control group and training group having identical descriptions of anticipated effects.

Another active control condition is one that incorporates non-adaptive versions of CCT. These programs use the same types of tasks as those in the experimental training condition, though they do not scale in difficulty with participant performance. When compared with adaptive versions of the same procedure, the tasks are balanced on participant expectancy and motivation, and practice effects can be ruled out. However, scaling difficulty introduces novelty, both in terms of activities encountered and strategies necessary for completing the task. Further, participants who complete the same task at unchanging difficulty may become less engaged as they reach their peak performance early. Controlling for practice effects is particularly important in older adults with cognitive impairment, given their propensity toward greater practice effects than their cognitively-stable peers (Suchy et al., 2011).

Remedial skills training has also been used as an active control condition. In these groups, participants discuss top-down strategies aimed at compensating with cognitive deficits. Such designs make it difficult to disentangle the plasticity of representations (knowledge, skills) from plasticity of processes (cognitive ability, brain function, brain structure, Fissler et al., 2015). It is possible that improvement following CCT is due to familiarity with neuropsychological tests and not due to improvement in underlying cognitive capacities. For example, learning to chunk numbers may improve digit span score, but may reflect one's ability to apply knowledge of cognitive strategies rather than one's actual attentional capacity. Comparisons between cognitive remediation and CCT are useful for balancing expectancy, motivation, and novelty, but offer little insight into the precise mechanism of any observed cognitive improvement.

## Looking forward

Future studies would benefit from inclusion of measures to evaluate non-specific factors as covariates. For example, the User Engagement Scale measures aspects of engagement, usability, and satisfaction on a 5-point Likert scale and comprises both negative (“I felt annoyed when on this site,” “the game was confusing”) and positive (“I really had fun,” “It was really worthwhile”) items (Wiebe et al., 2014). The Immersive Experience Questionnaire provides ratings of temporal distortion, challenge, emotional involvement, enjoyment, and attentional involvement in the task (Jennett et al., 2008). Expectancy can be evaluated using the Credibility and Expectancy Scale (Devilly and Borkovec, 2000). The CES asks participants to report how logical the therapy seems, how successful they expect the treatment to be, and whether they would recommend the treatment to a friend. Two factors, credibility, and expectancy, have been found within the CES, with expectancy ratings successfully predicting treatment outcome in a randomized controlled trial of cognitive therapies for generalized anxiety disorders.

Until CCT can be found to improve cognitive and everyday functioning after accounting for each non-specific factor, its future as a treatment remains uncertain. What is more certain is the usefulness of being generally mentally active. Indeed, high levels of mental activity may be associated not only with higher cognitive performance, but reduced risk of dementia. A systematic review of 22 population studies found mental exercises may reduce overall incident dementia risk by 46% (Valenzuela and Sachdev, 2006). Whether CCT is the same as any other form of mental activity or represents a unique method for augmenting cognitive processes shall be a continuing topic of investigation.

## Author contributions

JM: Manuscript conceptualization and design, writing of article. DD: Manuscript conceptualization and design, critical revision. PD: Manuscript conceptualization and design, critical revision. JS: Manuscript conceptualization and design, writing of article, critical revision. All authors have contributed to and have approved the final manuscript.

### Conflict of interest statement

PD has received grants and advisory/speaking fees from several pharmaceutical and technology companies including antidepressant manufacturers. He owns stock in several companies whose products are not discussed here. DD has received fees for scientific advisory boards from AbbVie, Lundbeck, and Intracellular Therapeutics. The other authors declare that the research was conducted in the absence of any commercial or financial relationships that could be construed as a potential conflict of interest.

## References

[B1] Au J. Sheehan E. Tsai N. Duncan G. J. Buschkuehl M. Jaeggi S. M. (2015). Improving fluid intelligence with training on working memory: a meta-analysis. Psychon. Bull. Rev. 22, 366–377. 10.3758/s13423-014-0699-x 25102926

[B2] Bandura A. (1982). Self-efficacy mechanism in human agency. Am. Psychol. 37, 122–147. 10.1037/0003-066X.37.2.122

[B3] Barnes D. E. Yaffe K. Belfor N. Jagust W. J. DeCarli C. Reed B. R.. (2009). Computer-based cognitive training for mild cognitive impairment: results from a pilot randomized, controlled trial. Alzheimer Dis. Assoc. Disord. 23, 205–210. 10.1097/WAD.0b013e31819c6137 19812460 PMC2760033

[B4] Boot W. R. Simons D. J. Stothart C. Stutts C. (2013). The pervasive problem with placebos in psychology: why active control groups are not sufficient to rule out placebo effects. Perspect. Psychol. Sci. 8, 445–454. 10.1177/1745691613491271 26173122

[B5] Carretti B. Borella E. Fostinelli S. Zavagnin M. (2013). Benefits of training working memory in amnestic mild cognitive impairment: specific and transfer effects. Int. Psychogeriatr. 25, 617–626. 10.1017/S1041610212002177 23253363

[B6] Clark R. Sugrue B. (1991). Research on instructional media, 1978-1988, in Instructional Technology: Past, Present, and Future, ed Anglin G.(Englewood, Colorado: Libraries Unlimited), 327–343.

[B7] Devilly G. J. Borkovec T. D. (2000). Psychometric properties of the credibility/expectancy questionnaire. J. Behav. Ther. Exp. Psychiatry 31, 73–86. 10.1016/S0005-7916(00)00012-4 11132119

[B8] Finn M. McDonald S. (2011). Computerised cognitive training for older persons with mild cognitive impairment: a pilot study using a randomised controlled trial design. Brain Impairment 12, 187–199. 10.1375/brim.12.3.187

[B9] Fissler P. Kolassa I. T. Schrader C. (2015). Educational games for brain health: revealing their unexplored potential through a neurocognitive approach. Front. Psychol. 6:1056. 10.3389/fpsyg.2015.01056 26257697 PMC4513287

[B10] Foroughi C. K. Monfort S. S. Paczynski M. McKnight P. E. Greenwood P. M. (2016). Placebo effects in cognitive training. Proc. Natl. Acad. Sci. U.S.A. 113, 7470–7474. 10.1073/pnas.1601243113 27325761 PMC4941515

[B11] Freeman T. B. Vawter D. E. Leaverton P. E. Godbold J. H. Hauser R. A. Goetz C. G.. (1999). Use of placebo surgery in controlled trials of a cellular-based therapy for Parkinson's disease. N. Engl. J. Med. 341, 988–992. 10.1056/nejm199909233411311 10498497

[B12] Gagnon L. G. Belleville S. (2012). Training of attentional control in mild cognitive impairment with executive deficits: results from a double-blind randomised controlled study. Neuropsychol. Rehabil. 22, 809–835. 10.1080/09602011.2012.691044 22712452

[B13] Gaitan A. Garolera M. Cerulla N. Chico G. Rodriguez-Querol M. Canela-Soler J. (2013). Efficacy of an adjunctive computer-based cognitive training program in amnestic mild cognitive impairment and Alzheimer's disease: a single-blind, randomized clinical trial. Int. J. Geriatr. Psychiatry 28, 91–99. 10.1002/gps.3794 22473855

[B14] Galante E. Venturini G. Fiaccadori C. (2007). Computer-based cognitive intervention for dementia: preliminary results of a randomized clinical trial. G. Ital. Med. Lav. Ergon. 29, B26–B32. Available online at: https://www.researchgate.net/publication/5279667_Computer-based_cognitive_intervention_for_dementia_Preliminary_results_of_a_randomized_clinical_trial 18575355

[B15] Gooding A. L. Choi J. Fiszdon J. M. Wilkins K. Kirwin P. D. van Dyck C. H.. (2016). Comparing three methods of computerised cognitive training for older adults with subclinical cognitive decline. Neuropsychol. Rehabil. 26, 810–821. 10.1080/09602011.2015.1118389 26674122

[B16] Goossens M. E. Vlaeyen J. W. Hidding A. Kole-Snijders A. Evers S. M. (2005). Treatment expectancy affects the outcome of cognitive-behavioral interventions in chronic pain. Clin. J. Pain 21, 18–26; discussion: 69–72. 10.1097/00002508-200501000-00003 15599128

[B17] Greenwood P. M. Parasuraman R. (2016). The mechanisms of far transfer from cognitive training: review and hypothesis. Neuropsychology 30, 742–755. 10.1037/neu0000235 26569030

[B18] Habib R. McIntosh A. R. Wheeler M. A. Tulving E. (2003). Memory encoding and hippocampally-based novelty/familiarity discrimination networks. Neuropsychologia 41, 271–279. 10.1016/S0028-3932(02)00160-4 12457753

[B19] Herrera C. Chambon C. Michel B. F. Paban V. Alescio-Lautier B. (2012). Positive effects of computer-based cognitive training in adults with mild cognitive impairment. Neuropsychologia 50, 1871–1881. 10.1016/j.neuropsychologia.2012.04.012 22525705

[B20] Horing B. Weimer K. Muth E. R. Enck P. (2014). Prediction of placebo responses: a systematic review of the literature. Front. Psychol. 5:1079. 10.3389/fpsyg.2014.01079 25324797 PMC4181242

[B21] Jennett C. Cox A. L. Cairns P. Dhoparee S. Epps A. Tijs T. (2008). Measuring and defining the experience of immersion in games. Int. J. Hum. Comput. Stud. 66, 641–661. 10.1016/j.ijhcs.2008.04.004

[B22] Karbach J. Verhaeghen P. (2014). Making working memory work: a meta-analysis of executive-control and working memory training in older adults. Psychol. Sci. 25, 2027–2037. 10.1177/0956797614548725 25298292 PMC4381540

[B23] Mangels J. A. Butterfield B. Lamb J. Good C. Dweck C. S. (2006). Why do beliefs about intelligence influence learning success? A social cognitive neuroscience model. Soc. Cogn. Affect. Neurosci. 1, 75–86. 10.1093/scan/nsl013 17392928 PMC1838571

[B24] Oken B. S. Flegal K. Zajdel D. Kishiyama S. Haas M. Peters D. (2008). Expectancy effect: impact of pill administration on cognitive performance in healthy seniors. J. Clin. Exp. Neuropsychol. 30, 7–17. 10.1080/13803390701775428 18165936

[B25] Petrovic P. Dietrich T. Fransson P. Andersson J. Carlsson K. Ingvar M. (2005). Placebo in emotional processing–induced expectations of anxiety relief activate a generalized modulatory network. Neuron 46, 957–969. 10.1016/j.neuron.2005.05.023 15953423

[B26] Rosen A. C. Sugiura L. Kramer J. H. Whitfield-Gabrieli S. Gabrieli J. D. (2011). Cognitive training changes hippocampal function in mild cognitive impairment: a pilot study. J. Alzheimers Dis. 26(Suppl. 3), 349–357. 10.3233/jad-2011-0009 21971474 PMC3277842

[B27] Rozzini L. Costardi D. Chilovi B. V. Franzoni S. Trabucchi M. Padovani A. (2007). Efficacy of cognitive rehabilitation in patients with mild cognitive impairment treated with cholinesterase inhibitors. Int. J. Geriatr. Psychiatry 22, 356–360. 10.1002/gps.1681 17117398

[B28] Rutherford B. R. Pott E. Tandler J. M. Wall M. M. Roose S. P. Lieberman J. A. (2014). Placebo response in antipsychotic clinical trials: a meta-analysis. JAMA Psychiatry 71, 1409–1421. 10.1001/jamapsychiatry.2014.1319 25321611 PMC4256120

[B29] Rutherford B. R. Sneed J. R. Tandler J. M. Rindskopf D. Peterson B. S. Roose S. P. (2011). Deconstructing pediatric depression trials: an analysis of the effects of expectancy and therapeutic contact. J. Am. Acad. Child Adolesc. Psychiatry 50, 782–795. 10.1016/j.jaac.2011.04.004 21784298 PMC3143372

[B30] Rutherford B. R. Wager T. D. Roose S. P. (2010). Expectancy and the treatment of depression: a review of experimental methodology and effects on patient outcome. Curr. Psychiatry Rev. 6, 1–10. 10.2174/157340010790596571 24812548 PMC4011659

[B31] Scott D. J. Stohler C. S. Egnatuk C. M. Wang H. Koeppe R. A. Zubieta J. K. (2007). Individual differences in reward responding explain placebo-induced expectations and effects. Neuron 55, 325–336. 10.1016/j.neuron.2007.06.028 17640532

[B32] Smeets R. J. Beelen S. Goossens M. E. Schouten E. G. Knottnerus J. A. Vlaeyen J. W. (2008). Treatment expectancy and credibility are associated with the outcome of both physical and cognitive-behavioral treatment in chronic low back pain. Clin. J. Pain 24, 305–315. 10.1097/AJP.0b013e318164aa75 18427229

[B33] Sneed J. R. Rutherford B. R. Rindskopf D. Lane D. T. Sackeim H. A. Roose S. P. (2008). Design makes a difference: a meta-analysis of antidepressant response rates in placebo-controlled versus comparator trials in late-life depression. Am. J. Geriatr. Psychiatry 16, 65–73. 10.1097/JGP.0b013e3181256b1d 17998306

[B34] Suchy Y. Kraybill M. L. Franchow E. (2011). Practice effect and beyond: reaction to novelty as an independent predictor of cognitive decline among older adults. J. Int. Neuropsychol. Soc. 17, 101–111. 10.1017/S135561771000130X 21073771

[B35] Talassi E. Guerreschi M. Feriani M. Fedi V. Bianchetti A. Trabucchi M. (2007). Effectiveness of a cognitive rehabilitation program in mild dementia (MD) and mild cognitive impairment (MCI): a case control study. Arch. Gerontol. Geriatr. 44(Suppl 1), 391–399. 10.1016/j.archger.2007.01.055 17317481

[B36] Tulving E. Kroll N. (1995). Novelty assessment in the brain and long-term memory encoding. Psychon. Bull. Rev. 2, 387–390. 10.3758/bf03210977 24203720

[B37] Valenzuela M. J. Sachdev P. (2006). Brain reserve and dementia: a systematic review. Psychol. Med. 36, 441–454. 10.1017/s0033291705006264 16207391

[B38] Wager T. D. Rilling J. K. Smith E. E. Sokolik A. Casey K. L. Davidson R. J.. (2004). Placebo-induced changes in FMRI in the anticipation and experience of pain. Science 303, 1162–1167. 10.1126/science.1093065 14976306

[B39] Wager T. D. Scott D. J. Zubieta J. K. (2007). Placebo effects on human mu-opioid activity during pain. Proc. Natl. Acad. Sci. U.S.A. 104, 11056–11061. 10.1073/pnas.0702413104 17578917 PMC1894566

[B40] Walsh B. T. Seidman S. N. Sysko R. Gould M. (2002). Placebo response in studies of major depression: variable, substantial, and growing. JAMA 287, 1840–1847. 10.1001/jama.287.14.1840 11939870

[B41] Weinberger J. (2014). Common factors are not so common and specific factors are not so specified: toward an inclusive integration of psychotherapy research. Psychotherapy 51, 514–518. 10.1037/a0037092 25111381

[B42] Wiebe E. N. Lamb A. Hardy M. Sharek D. (2014). Measuring engagement in video game-based environments: investigation of the user engagement scale. Comput. Hum. Behav. 32, 123–132. 10.1016/j.chb.2013.12.001

